# Altering the trajectory of early postnatal cortical development can lead to structural and behavioural features of autism

**DOI:** 10.1186/1471-2202-11-102

**Published:** 2010-08-19

**Authors:** Taylor Chomiak, Vikram Karnik, Edward Block, Bin Hu

**Affiliations:** 1 Department of Clinical Neurosciences, Hotchkiss Brain Institute, Faculty of Medicine, University of Calgary, Calgary, Alberta, T2N 4N1, Canada; 2 Department of Psychology, Mount Royal University, Calgary, Alberta, T3E 6K6, Canada

## Abstract

**Background:**

Autism is a behaviourally defined neurodevelopmental disorder with unknown etiology. Recent studies in autistic children consistently point to neuropathological and functional abnormalities in the temporal association cortex (TeA) and its associated structures. It has been proposed that the trajectory of postnatal development in these regions may undergo accelerated maturational alterations that predominantly affect sensory recognition and social interaction. Indeed, the temporal association regions that are important for sensory recognition and social interaction are one of the last regions to mature suggesting a potential vulnerability to early maturation. However, direct evaluation of the emerging hypothesis that an altered time course of early postnatal development can lead to an ASD phenotype remains lacking.

**Results:**

We used electrophysiological, histological, and behavioural techniques to investigate if the known neuronal maturational promoter valproate, similar to that in culture systems, can influence the normal developmental trajectory of TeA *in vivo*. Brain sections obtained from postnatal rat pups treated with VPA *in vivo *revealed that almost 40% of cortical cells in TeA prematurely exhibited adult-like intrinsic electrophysiological properties and that this was often associated with gross cortical hypertrophy and a reduced predisposition for social play behaviour.

**Conclusions:**

The co-manifestation of these functional, structural and behavioural features suggests that alteration of the developmental time course in certain high-order cortical networks may play an important role in the neurophysiological basis of autism.

## Background

Autism spectrum disorder (ASD) is a behaviourally defined brain disorder affecting approximately 1 in 150 children [1]. Autistic children exhibit impoverished verbal and non-verbal communication skills and reduced social interactions where they often bias their attention towards certain objects rather than the surrounding social situation [2]. Children with ASD also display behavioural impairments in attention engagement and disengagement, do poorly in emotional discrimination and facial recognition, and fail to response to their own names [2-6]. It has been suggested that behavioural phenotypes of ASD are associated with maturational changes in cortical thickness and organization, particularly affecting pyramidal neurons [1,7]. In addition, structural and functional abnormalities are particularly prominent in the temporal neocortex [1,8-10], and associated target structures including the amygdala [3], that mediate auditory and visual object recognition and attention orientation [1,11-14].

The underlying cellular and neurobiological mechanism(s) associated with ASD have remained elusive. Based on the work in autistic children, Susan Bryson has proposed that the expression of autistic behaviours may involve a hypersensitivity to sensory stimulation [6]. Indeed, recent work using one rodent model of autism has provided some evidence to support this conjecture. For example, Markram's lab has shown that rats prenatally exposed to VPA frequently exhibit hyper-connectivity and enhanced plasticity in prefrontal neocortical networks [15,16]. Hence, from a cellular level, increased neural activity in cortical networks may lead to abnormally noisy networks thus making it difficult for neural processing of certain sensory stimuli in the autistic brain [17].

The postnatal maturation trajectory of the neocortex is highly heterogeneous, exhibiting large regional variability in both structure and functional development [18-22]. This issue is, however, rarely addressed in the literature despite the fact that there is a growing realization that some of the key brain abnormalities of autism can be highly protracted and continue to evolve during postnatal life [23,24]. This is not surprising given the fact that certain high-order brain regions important for social functions endure continued plastic changes and delayed postnatal maturation [25,26]. For example, unlike some regions of the primary sensory and motor cortices, the speed of cortical maturation in high-order temporal association networks is significantly slower, often extending into adolescence [18-22]. This developmental feature suggests that the trajectory of temporal lobe development may be particularly sensitive to pathogenic factors that can influence the speed of neuronal maturation, especially during postnatal life [27]. For example, culture work has shown that valproate (VPA), and analogous compounds, are potent epigenetic factors that can facilitate neuronal maturation in neurons [28-30]. However, whether VPA can influence the speed of postnatal maturation *in vivo *and whether this can be associated with structural and behavioural characteristics related to autism remains unknown.

Here we address the emerging hypothesis that it may be the time course of postnatal cortical development that is most disturbed in ASD. To this end, we examined the TeA network from animals treated with a VPA dosage previously used *in vivo *[31]. We found that in addition to premature electrophysiological development of individual TeA cells, treated animals can exhibit gross cortical hypertrophy and a reduced predisposition for social play behaviour.

## Results

### Reduced social (play) interaction associated with VPA treatment

**Figure 1:**
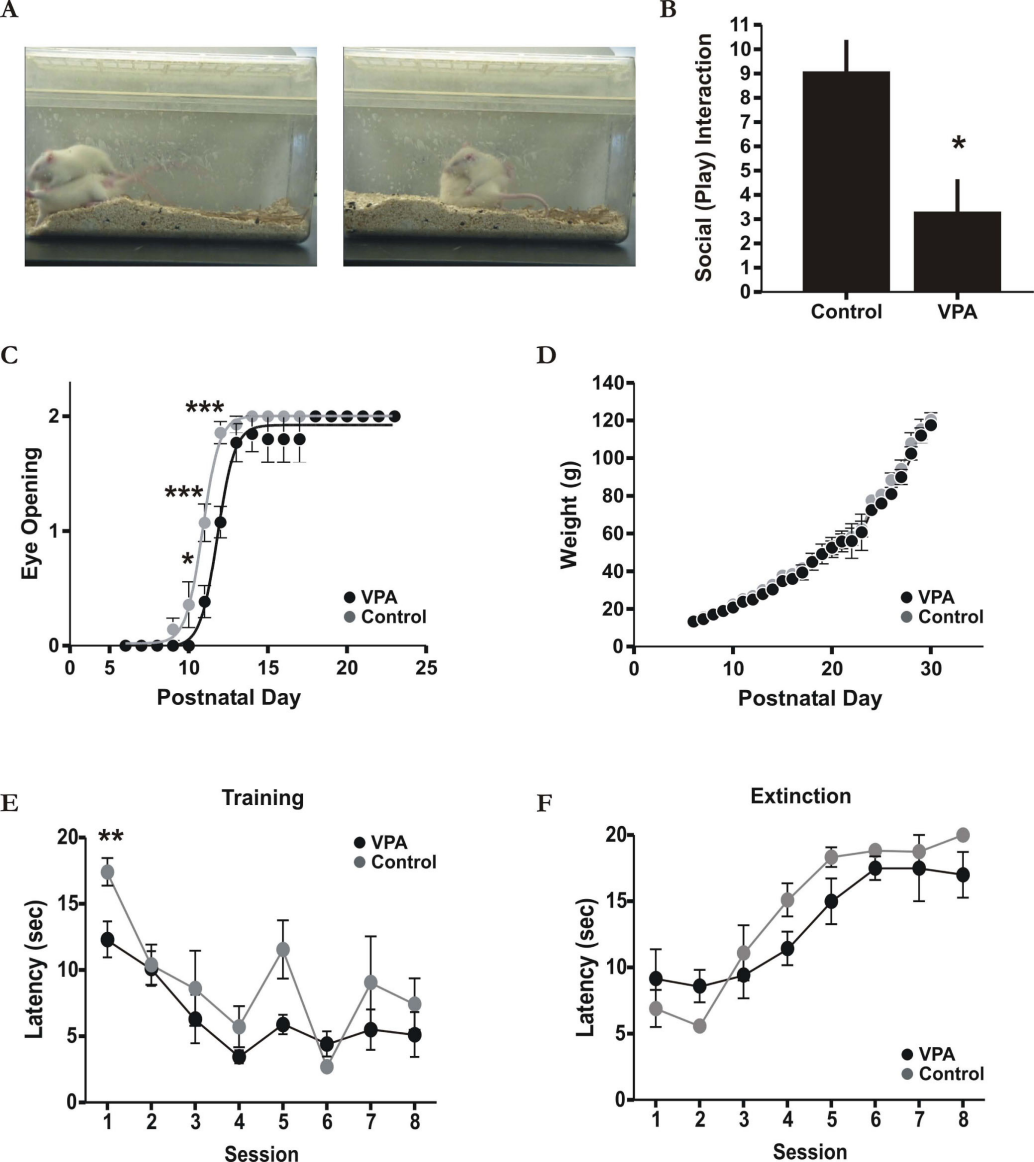
**Behavioural features associated with VPA-treatment**. A: Typical examples of rough-and-tumble play behaviours, a measure of play initiation and social interaction [32]. The left panel illustrates an "attack" (animal on top) to the nape and the right panel a "pin" by the rat. B: Summarized data of rough-and-tumble play behaviours scored in a ten minute test session between control and VPA-treated animals. * p < 0.05. C: significant delay of about 1-2 days (p < 0.05) in eye opening between control and VPA-treated animals. Eye scoring, similar to that defined previously [17], is as follows; 0 closed, 1 half-open, 2 complete eye-opening. D: Rat pup body weight between groups (i.e. control and VPA-treated) during the first month of life beginning on the first day of injection (P6). There was no obvious difference between groups (p > 0.05), an indicator of normal physical development under the rearing conditions for treated animals [34]. E: Cue-dependant associative learning for both VPA (black) and control (grey) animals. Rats were on a 47.5 h water deprivation schedule prior to training sessions. Animals were placed in the test cage and a 10 kHz sine-wave tone (5 pulses of 1 second in duration at 1 Hz) was present at random time points once the animal was in the opposite half of the cage relative to the reward location. Only when the 10 kHz tone was presented, was the reward (30% sucrose solution) available. Latency measurements represent the time between cue onset and arrival and orientation to the fixed reward location. F: Same protocol as in A, but now when the 10 kHz tone was presented, it was not reinforced (i.e. no reward was available). See Methods for more details. * p < 0.05; ** p < 0.01; ***p < 0.001.

The most prominent feature of autism is social impairment [2]. We therefore first examined whether VPA-treated animals also exhibited a similar behavioural pattern. We choose to investigate social play behaviour since it is one of the most widespread and least ambiguous forms of play amongst mammals [32]. As shown in Figure 1, we found a significant reduction in the number of rough-and-tumble play behaviours in VPA-treated animals as compared to age-matched (P35-37) controls (Figure 1A-B; Mann-Whitney *U *= 0; p < 0.05; n = 8, 2 sessions with 4 animals). For example, there was a reduction in the number of "attacks to the nape" and "pins" in treated rats relative to controls ([32] also see Methods). It is possible that reduced play behaviour following VPA-treatment may occur as a result of abnormal physical development under the rearing conditions for treated animals. To examine this, we first monitored eye opening time. As previously reported [17], we noticed a small but significant delay in eye opening by 1-2 days in treated rat pups (Figure 1C; two-way ANOVA Bonferroni post-test t ≥ 3.246 on day 10-12 and t ≤ 1.357 for all other days; p < 0.05; n = 14 and n = 17 for control and treated animals respectively), well before behavioural testing was undertaken. Second, we also measured changes in body weight between control and treated groups during the first month of life but did not observed any obvious difference between the two groups (Figure 1D; two-way ANOVA Bonferroni post-test t ≤ 1.244; p > 0.05; n = 14 and n = 17 for control and treated animals respectively). Finally, since VPA has previously been shown under some conditions to lead to generalized motor impairments in rodents [33], we tested to see if the reduced level of social play could be explained by general motor deficits. To this end, both VPA-treated and control animals underwent training and learned (two-way ANOVA F(7,152) = 13.2; p < 0.001) a cue-dependent reward-based sensorimotor task (see Methods for details). As shown in Figure 1E, VPA-treated animals were on par or even slightly better than controls at learning the association between the cue and the reward in addition to retrieving the reward (Figure 1E; two-way ANOVA Bonferroni post-test for session 1 t = 3.331 p < 0.01 and for all other sessions between treated and control groups t ≤ 2.265; n = 6 animals). Furthermore, extinction of the learnt task (two-way ANOVA F(7,106) = 16.99; p < 0.001) was also similar between controls and VPA-treated animals (Figure 1F; two-way ANOVA Bonferroni post-test t ≤ 1.774; p > 0.05). Taken together, these data indicate that the observed reduction in play behavior was unlikely due to general physical and/or rearing conditions [34]. Since rough-and-tumble play behaviour is a well-established measure of play initiation and social interaction [32], our data suggests a reduced predisposition for social interaction in VPA-treated animals.

### Enlarged temporal association cortex in VPA-treated animals

**Figure 2:**
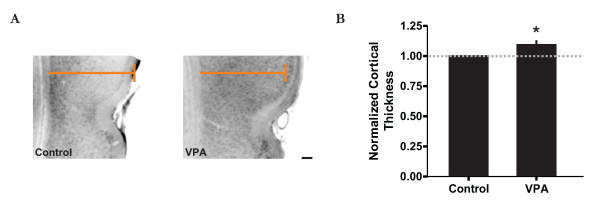
**Enlarged temporal association cortex in VPA-treated animals**. A: Images of the TeA area, located dorsal to the rhinal fissure (indent), for both control (left) and VPA-treated animals (right) illustrating an increase in cortical thickness in the latter. Note that the coloured line is identical for both images for reference. Scale bar = 200 μm. B: Normalized cortical thickness measurements of the TeA area between control and VPA-treated animals. * p < 0.05.

Since the temporal lobe is important in visual attention and social interaction [1,35,36], it is therefore not surprising that that many neuropathological findings with respect to the autistic brain are often associated with the temporal lobe region [8,9]. For example, evaluation of some autistic brains has revealed an increase in cortical grey matter and an enlargement of some regions of the temporal lobe by about 5-10% relative to typically developing controls [9,10]. Thus, to see if a similar histological pattern could be observed in our model, we examined TeA cortical grey matter thickness in VPA-treated and control animals similar to that done previously [37]. Although not clearly evident in all animals, we found a marginally significant increase in cortical thickness of just under 10% between age-matched (P20-23) treated and control animals (Figure 2; paired t-test t = 3.255; p < 0.05; n = 8 animals). Hence, this structural change in cortical anatomy resembles that observed in the human autistic brain, possibly reflecting an accelerated time course in cortical development [1].

### Accelerated development in the TeA network

**Figure 3:**
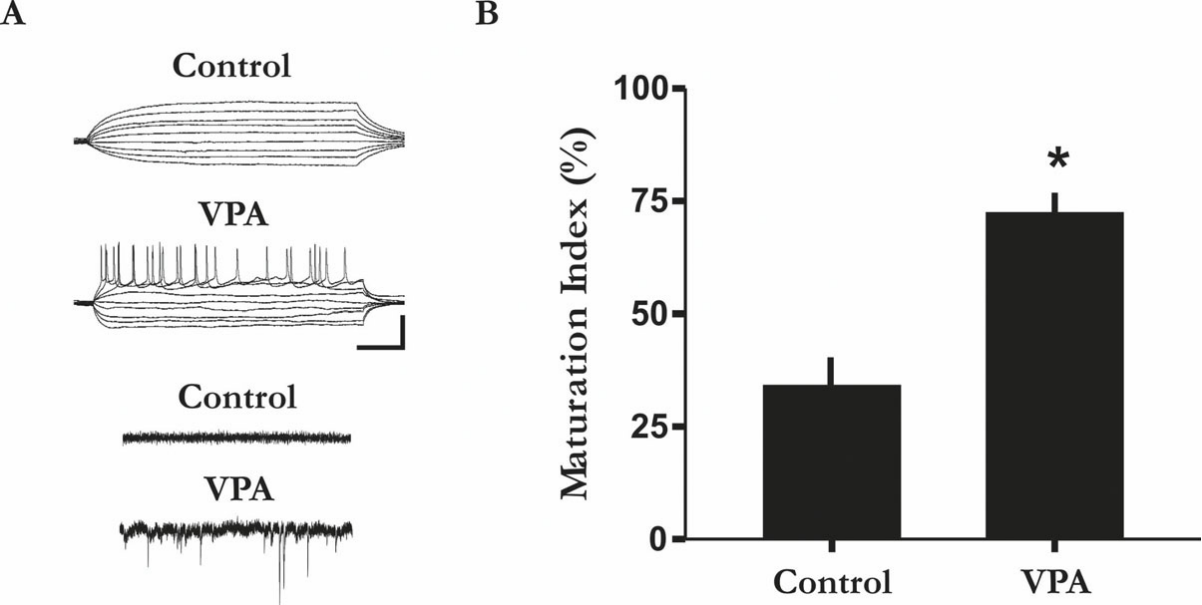
**Accelerated development of the TeA network in juvenile animals**. A: The majority of cells in treated animals exhibited adult-like electrophysiological properties and spontaneous synaptic activity ("VPA"), and is in stark contrast to that observed in saline or untreated control animals ("control"). The majority of these cells exhibited immature intrinsic neuronal properties similar to that found in new born rats [41]. Voltage responses to a current step protocol were applied from a holding or resting potential of around -60 to -70 mV. In voltage-clamp, cells were clamped at -60 mV. Sale bars: 30 mV vertical and 150 ms horizontal for top two traces; and 15 pA vertical and 400 ms horizontal for bottom two traces. B: Based on the intrinsic membrane properties (see panel A for example), juvenile (< 1 month) VPA-treated animals have a significantly higher maturational index as compared to juvenile control animals. *p < 0.05.

Previous work has shown that infragranular layers in the rat TeA consist of many slowly maturing pyramidal neurons [38]. Indeed, electrophysiological recordings have also shown that many infragranular TeA neurons in juvenile cortex (i.e., < 1 month) have not yet acquired adult-like intrinsic membrane properties (see Figure 3A for example) [39]. To examine the possibility of an accelerated time course of TeA development, we examined the intrinsic electrophysiological properties of individual cells which are known to undergo well characterized hallmark changes during development [40,41]. To this end, we obtained a total of 44 infragranular whole-cell recordings (n = 26 treated; n = 18 control) from juvenile VPA-treated and control animals. We found that the network contained significantly more cells with adult-like intrinsic neuronal properties in VPA-treated than saline or untreated control animals, including many neurons exhibiting intrinsic membrane excitability and spontaneous synaptic activities (Figure 3A). Using electrophysiological features as an index of neuronal maturation, we found that juvenile VPA-treated animals had a significantly higher number of maturing neurons as compared to control animals (Figure 3B; Mann-Whitney *U *= 0; p < 0.05). Hence, these data indicate a temporally accelerated pattern of neuronal development as a result of VPA-treatment.

### VPA does not appear to lead to cell death or the abnormal electrophysiological development of individual neurons

**Figure 4:**
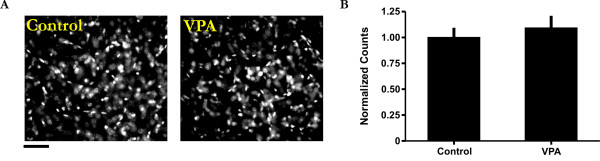
**Lack of cell loss due to VPA treatment**. A: Typical images of control and VPA-treated tissue sections illustrating a comparable pattern of DAPI staining. Scale bar = 50 μm. B: Summarized cell count data showing that there is no significant cell loss between control (n = 3 animals) and VPA-treated animals (p > 0.05; n = 3 animals). DAPI counts were conducted age-matched littermates.

**Figure 5:**
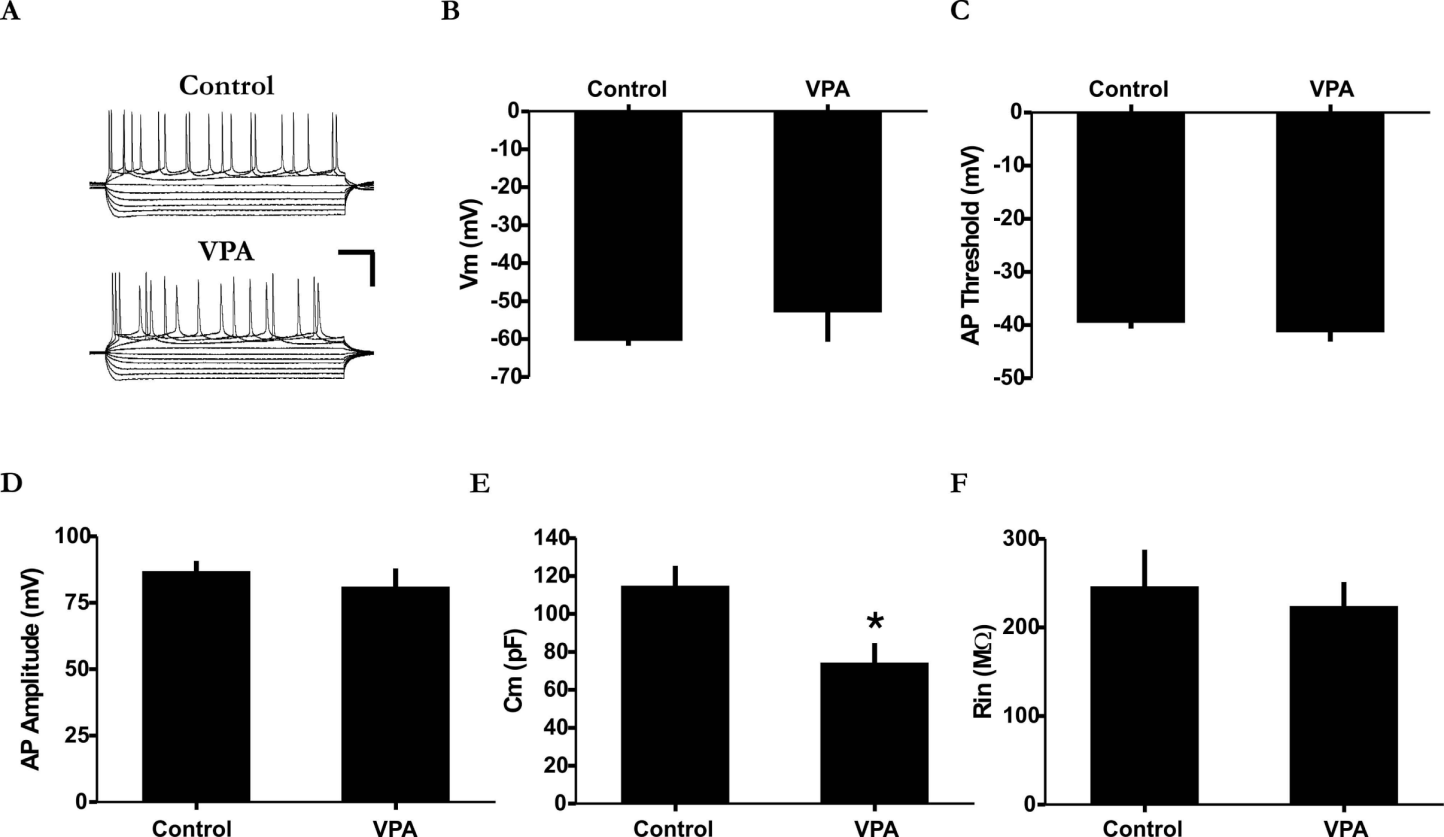
**Intrinsic membrane properties between control and VPA-treated neurons**. VPA-treatment does not appear to result in the abnormal electrophysiological development of TeA neurons. A: Recordings from a neuron obtained from a control (top) and VPA-treated animal (bottom) illustrating a similar voltage response to current injection. Scale bars = horizontal 100 ms; vertical 40 mV. B-F: Comparison of five basic intrinsic membrane properties. B, resting membrane potential; C, action potential threshold; D, action potential amplitude; E, membrane capacitance; and F, input resistance. The membrane capacitance was slightly smaller in neurons of VPA-treated animals although it is still significantly larger than immature neurons and comparable to those obtained from control animals (not shown). Data are from animals of similar age. * p < 0.05.

Although VPA treatment has been shown to promote neuronal maturation [28], its effect on cortical neurons may also involve tissue toxicity, cell injury and death [42]. To exclude these possibilities, we first conducted DAPI cell counts and found no evidence of cell loss due to VPA treatment (Figure 4; Mann-Whitney *U *= 4; p > 0.05; n = 6 animals). In fact, we noted a slight increase in cell number. In the second set of experiments we examined whether VPA can have a non-specific injury type of an effect on the membrane properties of TeA neurons. Of the five basic intrinsic membrane properties examined, all but one was similar between the two groups (Figure 5; membrane potential (V_m_), Mann-Whitney *U *= 17; spike threshold, Mann-Whitney *U *= 15; spike amplitude, Mann-Whitney *U *= 14; input resistance (R_in_), Mann-Whitney *U *= 16; n = 12 neurons). Only the membrane capacitance appeared to be smaller in treated animals relative to controls (Mann-Whitney *U *= 5; p < 0.05). However, this reduction can not be considered abnormal given the fact that it was still several-fold larger than immature neurons and comparable to some spiking cells in control animals (data not shown). Taken together, these data suggest that the effect of VPA on network and cellular development is unlikely due to deleterious effects on neuronal viability and functional integrity.

## Discussion

### Experimental approach

VPA has previously been associated with ASD based on the fact that children born to pregnant women taking VPA for seizure management had an elevated risk of developing ASD [15,43]. However, although some of the abnormalities observed in the autistic brain are of prenatal origin, postnatal factors are also considered to play an important role [23,27]. In fact, very little research has attempted to address how chemical agents may influence certain postnatal developmental processes [27]. In our model, the adverse effect of VPA may be derived from a persistent effect on the temporal lobe as it undergoes a protracted and delayed maturation. For example, VPA has been frequently used in infants to manage seizure [44]. Nevertheless, the importance of our experimental approach is that it has allowed us to directly examine the emerging hypothesis that it is an altered time course of brain development that is most disturbed in autism [1].

The timing of brain insult also seems to differ in terms of the phenotypes of ASD. For example, later exposure in humans has largely been associated with the nonsyndromic and sometimes high-functioning forms of ASD [45]. By contrast, early exposure to an insult (e.g. first trimester) represents the time when the syndromic (i.e., multiple congenital anomalies and mental retardation) form of ASD is thought to be initiated [45]. In this regard, the main physical developmental delay we observed here is a short delay in eye opening (see Figure 1) as reported previously [17]. However, in an exceptional case, we found one pup among all littermates that had a persistently low body weight and delayed fur growth during early postnatal development which was excluded from our analysis. Interestingly, a persistently low body weight was also noted in most (if not all) animals in a previous study using an early prenatal VPA treatment protocol [17]. Hence, the temporal difference between VPA treatment may produce certain biases related to behavioural and developmental phenotypes [33] in which later insults may more closely reflect nonsyndromic high-functioning forms of autism.

### Cortical structural and electrophysiological changes induced by VPA

The most consistent neuropathological finding with respect to the autistic brain is cortical enlargement, particularly in the temporal lobe region [8,9]. This is not surprising since the temporal lobe association networks are involved in many processes including attention, social interaction, object recognition, and biased competition where the object's neural representation dominates across multiple networks [1,11-13,35,46,47]. Post-mortem examination of some autistic brains has revealed an increase in cortical grey matter and hyper-convolution of some regions of the temporal lobe [8]. In particular, Piven and colleagues noted that the largest relative increase in cortical grey matter was in the temporal lobe [9]. Their value of around a 5% increase in the temporal lobe for typically developing controls is consistent with our results (Figure 2) and that of a recent study by Schumann and coworkers of around 10% [10].

The mechanism through which VPA influences neuronal development and cortical thickness is still not entirely clear and is likely complex and multifactorial. Although VPA is classically considered an anti-epileptic drug, a more recent study has shown that VPA appears to have a strong influence in mediating epigenetic modulation rather than enhanced GABA activity [31]. Indeed, in our study the accelerated appearance of adult-like membrane properties in VPA-treated animals was obtained at a dosage that has been shown to inhibit histone deacetylases *in vivo *and activate epigenetic signalling pathways in the rat [31]. These data indicate a possible role of chromatin remodelling in the control and execution of developmental gene programs involved in different aspects of neuronal maturation [28-30,48]. However, future study is required to determine whether potential changes to the intracellular somatodendritic lipid and/or protein organization which may be contributing to the immature electrophysiological phenotype can also be related to the emergence of mature intrinsic properties [49,50]. Nevertheless, *in vivo *VPA-treatment does appear to facilitate premature development of the TeA network. Interestingly however, inactivation of PTEN signalling mechanisms can also lead to increased cortical thickness [37], while mutations leading to the formation of tuberous sclerosis complex, which is strongly associated with ASD [51], can also result in more active cortical networks in both humans and rodents [52-54]. Thus, VPA may exert its effect on neuronal development and cortical anatomy through activation of gene programs related to activity development [29] and/or activity-dependent processes and signal transduction mechanisms [37,55,56].

### VPA-induced behavioural changes

As noted by Leo Kanner in 1943, autistic children tend to avoid social interaction [2]. Over the past several years, different indices of social interaction in animal models have been investigated and are consistently found to be reduced [17,37,57]. Social play behaviour has been well documented in the rat and is also considered one of the most widespread and least ambiguous forms of play amongst mammals [32]. However, this type of social interaction, to our knowledge, has never previously been evaluated in relation to an autistic phenotype under these conditions.

It is well recognized that high-order temporal lobe networks play a critical role in auditory and visual processing, attention, and ultimately, social interaction [1,11-14,35,36,47,58-60]. Autistic children often have difficulty with attention disengagement [5,6] and frequently fail to react to their names so much so that parents often suspect deafness [61]. Hence, the reduced social play behaviour we report here may reflect complex functional impairments in multiple sensorimotor domains. Indeed, a recent study has shown that the TeA network in rats can form a descending reticular-like network, providing top-down innervations to many cortical and subcortical structures including the amygdala [46]. Thus, premature development of even a small population of TeA neurons may have a powerful influence on many downstream structures that are also involved in working memory, attention, and emotional behaviours.

## Conclusions

The main finding of the present study indicates that early postnatal exposure to VPA, at a dosage known to inhibit histone deacetylases *in vivo *[31], can lead to synchronous developmental alterations reminiscent of ASD. Neurons from VPA-treated animals tended to acquired adult-like electrophysiological properties sooner during early postnatal development relative to controls. Furthermore, these electrophysiological changes were also associated with an increase in cortical thickness and a reduced predisposition for social interaction. Such a co-manifestation of features is consistent with the emerging theory that it is the time course of early postnatal development that may be most disturbed in ASD [1].

## Methods

### Preparation and electrophysiology

All experimental protocols were approved by the University of Calgary Conjoint Faculties Research Ethics Board. Under this protocol, animals are housed in The University of Calgary Animal Resource Center facility receiving constant care throughout the year. Briefly, newborn male and female Sprague-Dawley rats were socially caged after weaning at day P21. Frontal tissue sections were prepared on a Leica vibrotome (Germany). Slices (≈300 μm) were submerged in a recording chamber in oxygenated (95% 0_2_; 5% CO_2_) artificial cerebrospinal fluid (aCSF). The aCSF had a final pH and osmolality of around 7.4 and 290-300 mOsm/kg respectively and contained (in mM): NaCl, 110; KCl, 3.5; MgCl_2_, 1.5; NaHCO_3_, 26; CaCl_2_, 2 and glucose, 10. The patch electrode solution contained (in mM): K-gluconate, 120; KCl, 10; Na-HEPES, 10; Na-GTP, 0.2; Na-ATP, 4. Whole-cell patch-clamp recordings were targeted to layer V of the posterior sector of the TeA-ectorhinal cortex (also known as TeV). The TeA region is identified, under DIC microscopy, as the region located dorsally to the rhinal fissure [62,63]. The patch electrode solution had an osmolality and pH of around 285 mOsm and 7.2 respectively. The DC resistance of patch electrodes was 6-8 MΩ and recordings were made at 30-33°C. The bridge-balance and liquid-junction potential were corrected on-line. Intrinsic membrane properties were determined according to methods previously described [64].

### *in vivo *valproate injections

The sodium salt of VPA (NaVPA; Sigma-Aldrich, St. Louis, MO) was dissolved in 0.9% saline (pH ≈7.3). Treated rats received a single i.p. injection on the order of 150 mg/kg/day [31]. It should be noted that due to the species variation in valproate metabolism, direct dosage correlations can not be made with humans [65]. However, from these data, we may predict that the functional dose in rat models of seizure would be slightly higher than that of humans, which is indeed the case [42,44,66]. Injections started on P6 and then continued for a maximum of 2 weeks. We also injected littermate control rats with 0.9% saline (pH ≈7.3). Recordings were made from juvenile (< 1 month) animals ranging from P12-P26.

### Social (play) behaviour

Animals were isolated in macrolone cages measuring 43 cm × 15 cm × 28 cm for a period of 24 hours prior to testing similar to previously described [17]. The test consisted of placing two rats (either saline injected or VPA injected) into the test cage for a period of 10 minutes. The animals (male/female pairs) were tested at the same age (i.e., age-matched littermates ranging from P35-P37) and weight (±20 g) as each other. Rough-and-tumble play behaviors (e.g., a direct "attack" to the nape on the neck of the other rat or "pinning" where one of the animals is lying with its dorsal surface on the floor of the test cage with the other animal standing over them), were measured as an indicator of social interaction [32].

### Cued sensorimotor task

A total of 6 male and female animals (3 pairs; sex and age-match littermates) were used. Animals were water deprived for 47.5 h prior to behavioural testing but had unrestricted access to food. Following each session, animals had unrestricted access to water for 0.5 h. Training sessions consisted of ≥3 trials and began on postnatal day 23-30. Training occurred in the same test cage with an approximate 3-5 min break between trials. This also allowed for the paper towel bedding of the test cage to be replaced following each trial. The experiments were conducted in an isolated quiet room or sound-attenuating chamber and the location did not change once training had begun. The test cage consisted of a macrolone cage with a hole drilled a few centimeters from the bottom at one end (fixed reward location) just large enough to permit insertion of a water bottle spout. The wall was covered so the animal could not see the bottle contents that the spout was attached to. Latency measurements represent the time difference between auditory cue presentation and arrival at, and orientation to, the fixed reward location. A maximum cut-off of 20 seconds was used. A spout was always present but only when the cue was presented and the animal arrival at and orientated to the fixed reward location was the 30% sucrose solution available. A computer with a National Instruments A/D board (NI DAC-Card 6024E, 200 kSamples/s, 16 channels), a breakout box (National Instruments BNC-2020), and a high speed IEEE 1394a port was used to run in-house software to collect timing information. A simple on/off switch was connected to the computer to trigger synchronization between the tone output and latency measurements. The software generated a 10 kHz 80-85 dB sin wave output played through speakers placed at a short and fixed distance from the cage. The software recorded the button press, started the audio signal, and recorded the second button press when the rat touched the reward (i.e. water bottle spout). The time from the first button press to the second was logged in a data file and presented as a temporal latency.

### Cortical thickness measurements and DAPI staining

Age-matched littermate brains from juvenile animals (e.g. P20-P23) were removed and fixed in 4% PFA overnight at 4°C. Frontal sections (150 μm) were then cut on a Leica vibrotome (Germany) in PBS. Images were acquired with a Zeiss microscope (Zeiss Axioplan Fluorescence Microscope; Carl Zeiss, Germany) and analyzed off-line. Cortical thickness measurements (i.e. distance between the underlying white matter to the pia) were made similar to that reported elsewhere [37]. To reduce the potential for a masking effect of relatively small differences by the large variability between littermates, data was normalized to animals from the same litter. For 4',6-diamidino-2-phenylindole (DAPI) staining, sections were counterstained with DAPI (1:10^4^) for 5 min. at room temperature followed by a 1 × PBS rinse 3 times for 5 min. Deep layer TeA cell counts corresponded to the region of electrophysiological recordings.

### Analysis

Signal acquisition and analysis was accomplished using Multi-clamp 700A and DIGIDATA 1322A 16-Bit data acquisition system and Clampex 9 programs allowing for data to be low-pass filtered at 2-8 kHz and digitized at ≥10 kHz (Axon Instruments, Inc. Forster City, CA, USA). Images analysis was accomplished using ImageJ software (NIH). Data are expressed as mean ± SEM. The statistical test and p value are noted when used. Two-tailed tests were used if not otherwise stated. Statistical analysis was accomplished with GraphPad software.

## Authors' contributions

Conceived and designed the experiments: TC/BH. Developed and performed the experiments: TC/VK/EB. Analyzed the data: TC/VK. Wrote the paper: TC/BH. All authors read and approved the final manuscript.
